# Clonal hematopoiesis with *TET2* mutations spawning synchronous primary central nervous system lymphoma and myelodysplastic syndrome

**DOI:** 10.1007/s00277-023-05430-z

**Published:** 2023-09-13

**Authors:** Hannes Treiber, Christina Ganster, Julie Schanz, Joji Shimono, Sabrina Zechel, Nilofar Pohanyar, Christian Riedel, Christine Stadelmann, Detlef Haase, Lorenz Trümper, Björn Chapuy, Gerald G. Wulf

**Affiliations:** 1 Department of Hematology and Medical Oncology, University Medical Center Göttingen, Robert-Koch Str. 40, 37075 Göttingen, Germany; 2 Department of Neuropathology, University Medical Center Göttingen, Göttingen, Germany; 3 Department of Neuroradiology, University Medical Center Göttingen, Göttingen, Germany; 4 Department of Hematology, Oncology and Tumor Immunology, Charité Universitätsmedizin Berlin, Campus Benjamin Franklin, Berlin, Germany

Dear Editor,

Clonal hematopoiesis (CH) describes the aging-related phenomenon of clonal segregation in hematopoiesis driven by acquired mutations. CH is not only found in healthy individuals but is also associated with increased incidences of hematopoietic neoplasms. Loss-of-function mutations in Ten-Eleven Translocation 2 (*TET2*) represent the second most frequent CH finding, and also occur in 10% of diffuse large B-cell lymphomas (DLBCL). We report a patient in whom mature B- and T-cells and cells of a myelodysplastic syndrome (MDS) and a primary central nervous system lymphoma (PCNSL) shared two identical *TET2* mutations, suggesting a divergent evolution of both from a common CH precursor.

**Fig. 1:**
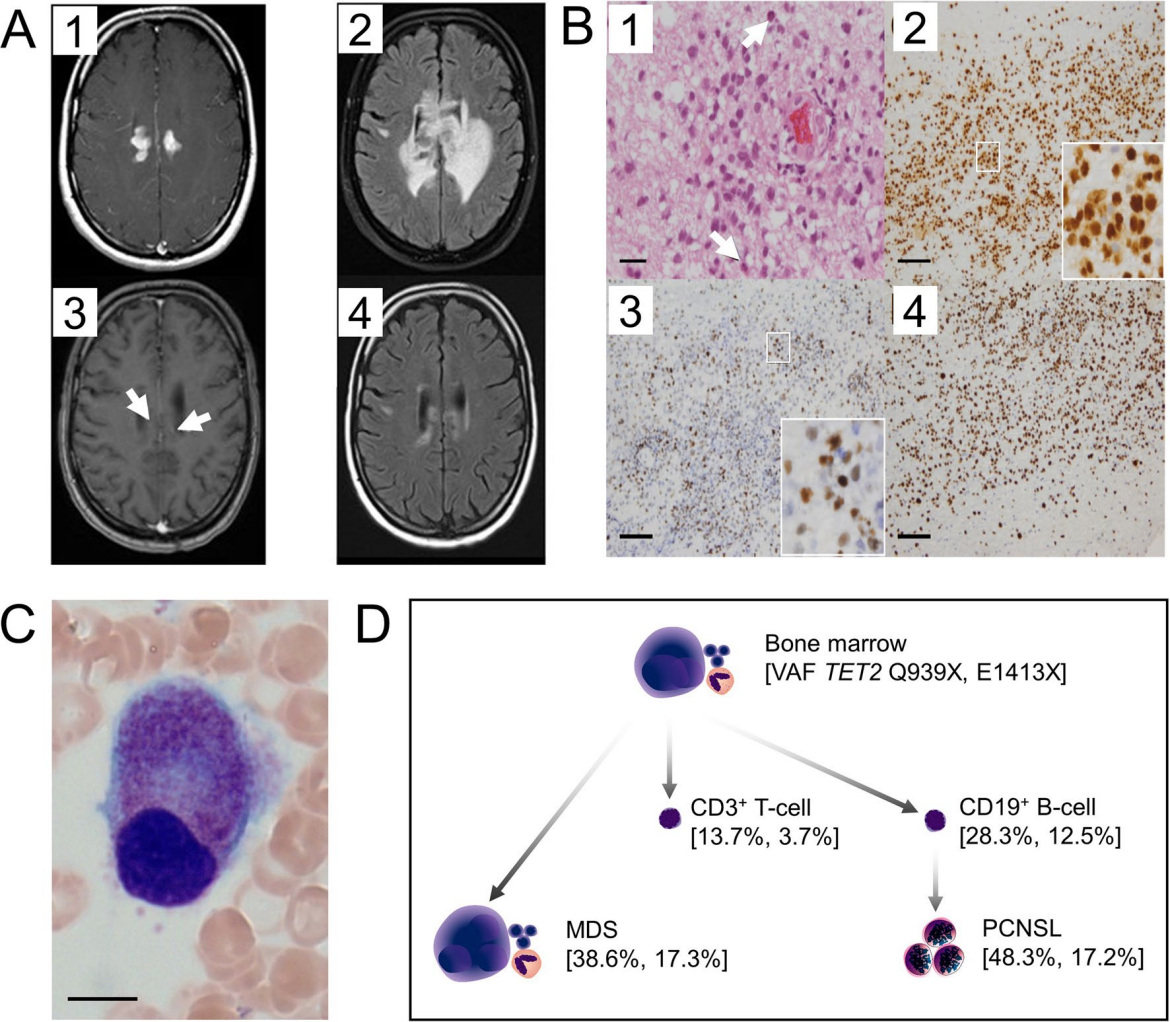
Clinical and molecular features of the *TET2* mutated PCNSL case. **A** Cerebral magnetic resonance imaging. 1–2: axial T1-weighted and FLAIR images demonstrated strong contrast enhancement and perifocal edema; 3–4: regression of PCNSL by one course of MATRIx immunochemotherapy: only two small spots (arrows) with contrast enhancement remain in the ventricular wall. **B** PCNSL histology: 1: HE-stained section of brain biopsy showed distinct infiltrates of aggressive B-cell lymphoma (arrow heads: cells undergoing mitosis); 2–3: expression of B-cell markers PAX5 (2) and BCL6 (3). 4: High proliferation index indicated by Ki67 staining. Scale bars: (1): 20 µM, (2)–(4): 100 µM. **C** MDS cytology: cytology of bone marrow sample showing a dysplastic megakaryocyte. Pappenheim’s staining, 1000 × . Scale bar: 10 µM. **D** Evolutionary tree of *TET2* mutations found in bone marrow, sorted CD3 + T- and CD19 + B-cells and the PCNSL specimen. Indicated are VAFs for *TET2* mutations Q939X (1st VAF) and E1413X (2nd VAF) for the respective sample

In a 60-year-old female with headaches, magnetic resonance imaging revealed an intracerebral mass (Fig. 1A). Stereotactic biopsy and staging including bone marrow biopsy with normal cellularity confirmed the diagnosis of PCNSL (Fig. 1B). After one course of immunochemotherapy with MATRix, hematological regeneration was delayed. Repeat bone marrow biopsy revealed dysplastic megakaryopoiesis and granulopoiesis, suspicious of MDS (Fig. 1C). NGS analysis covering 49 MDS/CHIP-related genes was applied to samples from bone marrow, FACS-sorted peripheral blood B- and T-cells, and the PCNSL. With a mean target coverage of 262 and 282 reads in the PCNSL and 540 to 1529 reads in the other specimen, all samples harbored two *TET2* mutations (Q939X and E1413X) (Fig. 1D). Somatic origin was confirmed by fingernail analysis. Both mutations likely lead to loss-of-function by premature stop codons. While the chemotherapy had induced partial remission of the PCNSL, cytopenia prevented further cytotoxic treatment. Therefore, whole brain radiation therapy was applied, inducing complete remission, ongoing 34 months after diagnosis. At 28 months, transfusion-dependent thrombocytopenia occurred, without evidence for cytogenetic or molecular MDS progression. Thrombopoietin-agonist therapy succeeded in stabilization of platelet counts.

This case reflects divergent evolution of a CH-derived progenitor to an MDS and a PCNSL. While inactivating *TET2* mutations represent characteristic findings in CH/MDS, they are not frequent in PCNSL [1–4]. PCNSL lymphomagenesis is fueled by inactivation of *CDKN2A* and co-occurring oncogenic mutations in *CD79B* and *MYD88*^L265P^, similar to DLBCL genetic subset C5/MCD [2, 5, 6]. Accordingly, *TET2* mutations are usually not found in C5/MCD DLBCL, but TET proteins have multiple functions in early and late B-cell development [7], possibly contributing to lymphomagenesis of this case. Less likely, the transforming events have hit a CH B-cell progenitor without a specific role of the *TET2* mutations (passenger mutations). Divergent evolution of CH to myeloid neoplasms and T-cell lymphomas was demonstrated in two independent reports [8, 9]. Intriguingly, lineage analysis of CH mutations in larger series revealed higher variant allele fractions of *TET2* mutations in B- than in T-cells [10], consistent with our observation (Fig. 1D). Regardless of their mechanistic role, the *TET2* mutations inform on the timing of PCNSL formation in this case: they must have preceded the B-cell transformation towards PCNSL.

In summary, this is the first description of PCNSL and MDS emanating from a common *TET2* mutated progenitor. Future genomic studies of larger PCNSL cohorts as well as functional analysis may elucidate the role of *TET* mutations in PCNSL biology.
